# Supplementary figures and images for: Linking germline telomere removal to global programmed DNA elimination in Tetrahymena genome differentiation
Source: eLife. 2026 Jul 14;15:RP109351. doi: 10.7554/eLife.109351 (PMC13368176; doi:10.7554/eLife.109351)

**Fig. 5D**

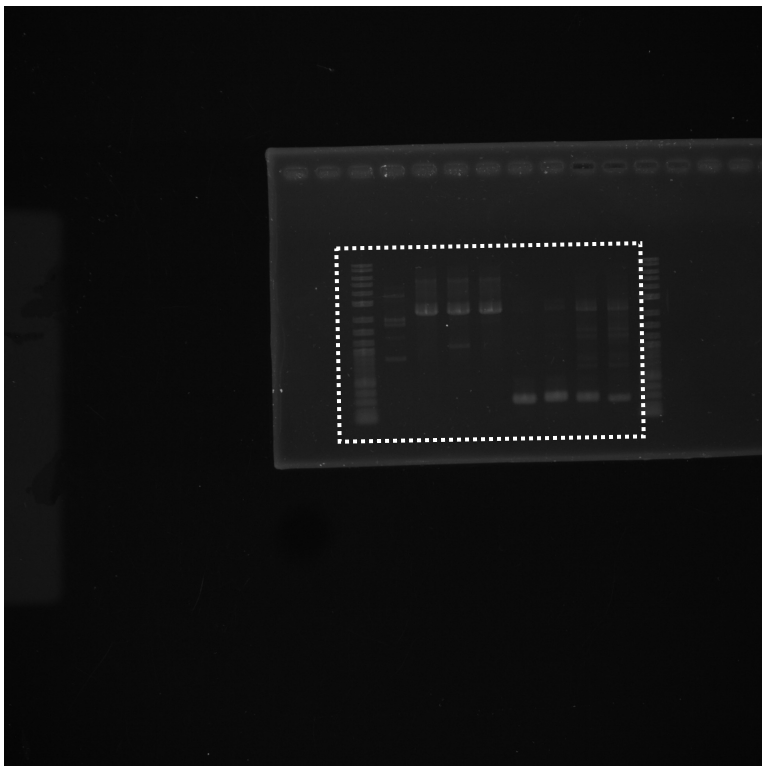

**Fig. 5E**

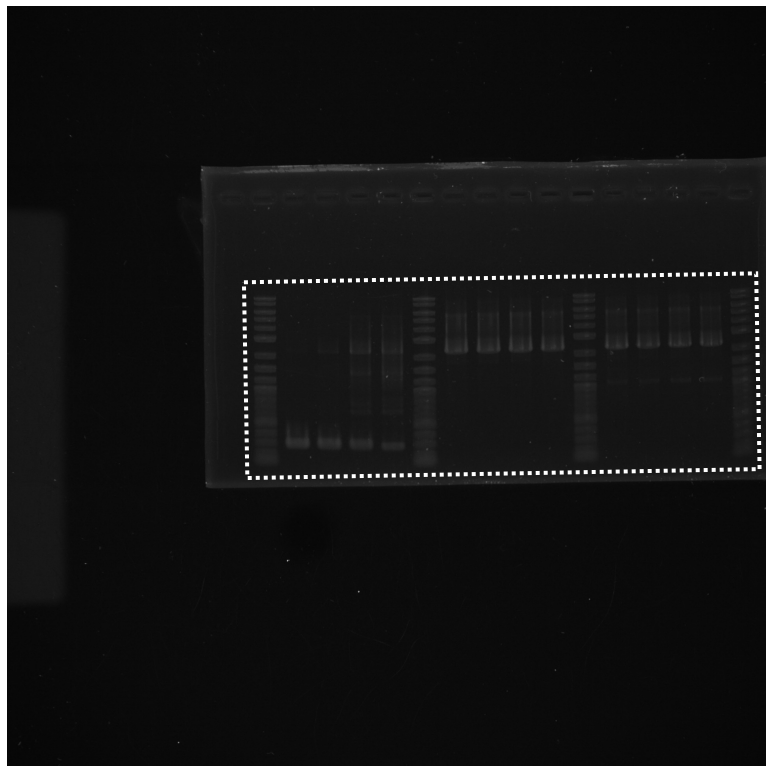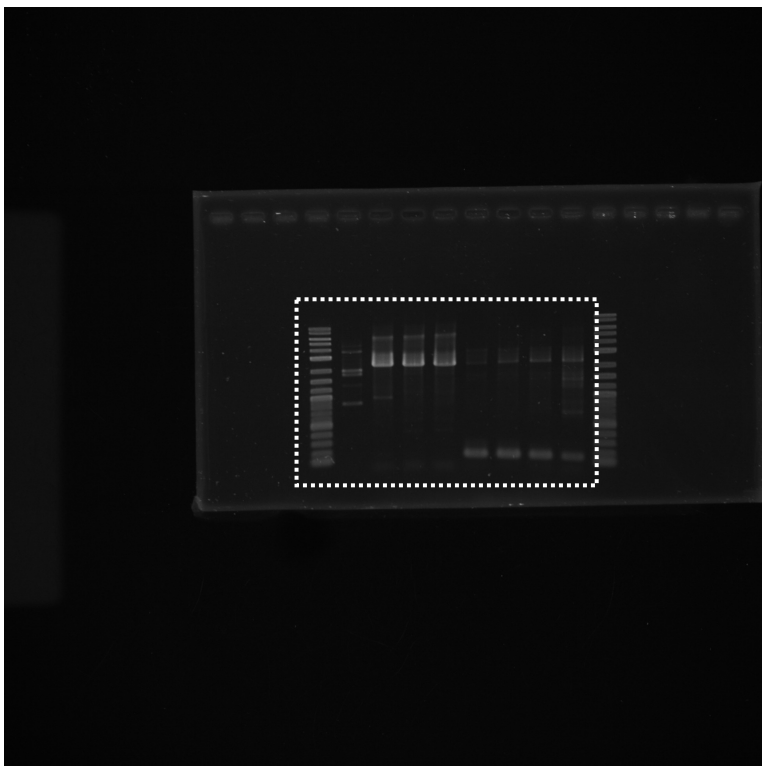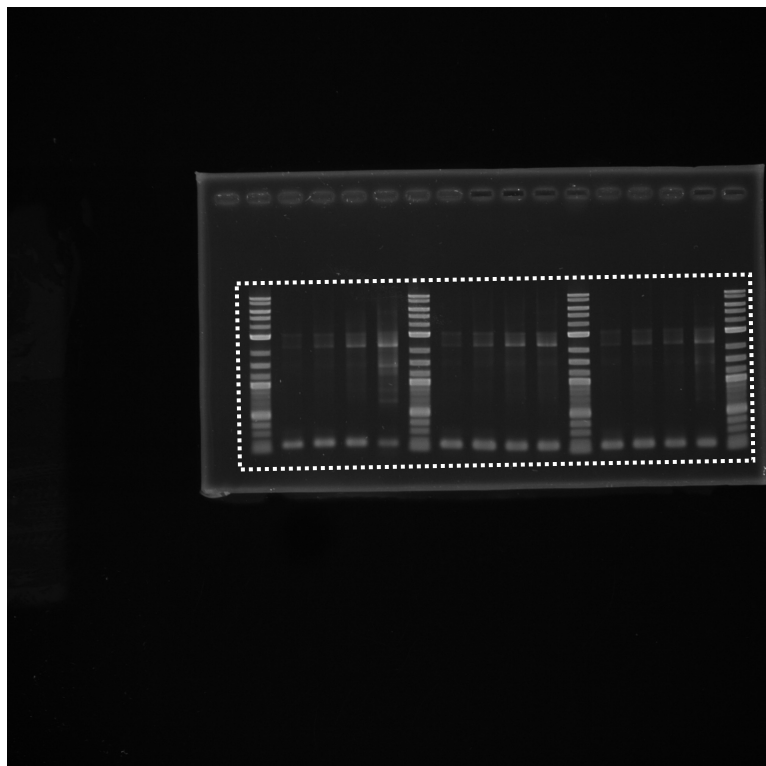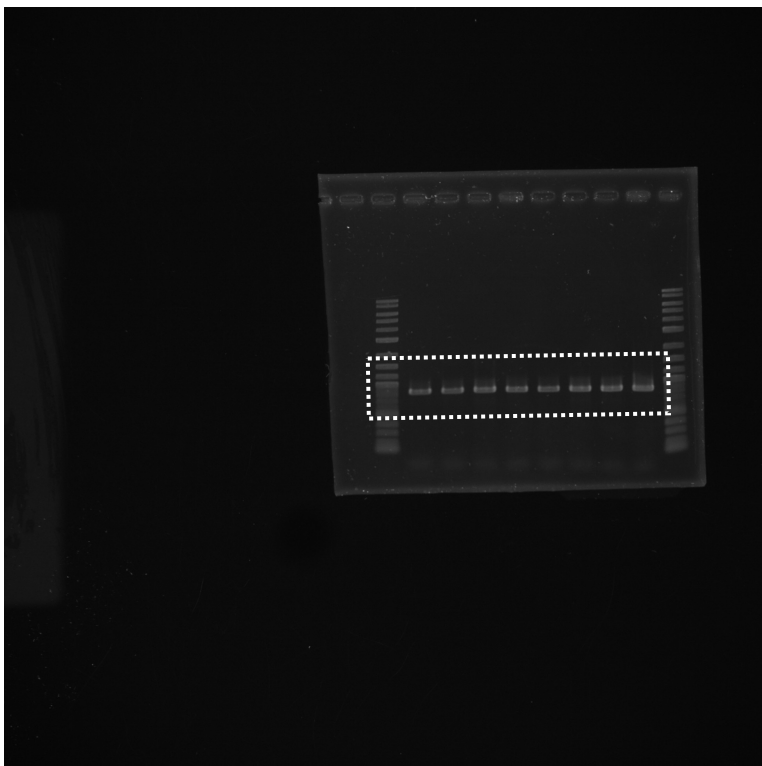

Supplement: Figure 5—source data 1. [file elife-109351-fig5-data1.pdf]
