# Supplementary material for: Linking germline telomere removal to global programmed DNA elimination in Tetrahymena genome differentiation
Source: eLife. 2026 Jul 14;15:RP109351. doi: 10.7554/eLife.109351 (PMC13368176; doi:10.7554/eLife.109351)
Supplement: Supplementary file 1. [file elife-109351-supp1.docx]

| MIC Chromosome end | MLS length (nt) | Unique 32 nt Sequences | Unique 32 nt Sequences with 40-70% GC | Non-overlapping 32 nt Probes |
| --- | --- | --- | --- | --- |
| 1L | 431352 | 140701(32.6%) | 8473 (2.0%) | 596 |
| 2L | 276442 | 95365 (34.5%) | 5199 (1.9%) | 373 |
| 2R | 289068 | 63154 (21.9%) | 3829 (1.3%) | 296 |
| 3L | 441828 | 98378 (22.3%) | 6237 (1.4%) | 469 |
| 3R | 438933 | 72024 (16.4%) | 3906 (0.9%) | 275 |
| 4L | 286856 | 46536 (16.2%) | 2690 (0.9%) | 223 |
| 4R | 463156 | 95159 (20.6%) | 5365 (1.2%) | 423 |
| 5L | 276432 | 54218 (19.6%) | 3168 (1.1%) | 238 |

**Table A. Designing MLS-specific Oligo Probes**
