# Supplementary material for: Linking germline telomere removal to global programmed DNA elimination in Tetrahymena genome differentiation
Source: eLife. 2026 Jul 14;15:RP109351. doi: 10.7554/eLife.109351 (PMC13368176; doi:10.7554/eLife.109351)
Supplement: Peer review history

# Editor report: eLife Assessment

**DOI:** 10.7554/eLife.109351.3.sa0

**Authors:** Sigurd J Braun

**Affiliations:** Justus-Liebig University Giessen Germany

**Keywords:** Compelling, Important

This **important** study reveals intriguing connections between chromosome breakage and DNA elimination during programmed genome rearrangement in the ciliate *Tetrahymena thermophila*. By developing a novel FISH approach that distinguishes germline and somatic telomeres, the authors provide **compelling** evidence that chromosome breakage removes germline telomeres along with hundreds of kilobases of germline-limited sequences. By disrupting a single chromosome breakage site, they further showed that DNA elimination was globally affected, which opens up a new direction for mechanistic studies. Thus, this work reveals additional similarity between the programmed DNA elimination in ciliates and nematodes that underlies the transition from germline to somatic telomeres.

# Referee report: Reviewer #1 (Public review)

**DOI:** 10.7554/eLife.109351.3.sa1

Summary:

In this study entitled "Linking Germline Telomere Removal to Global Programmed DNA Elimination in Tetrahymena Genome Differentiation" Nagao and colleagues examine the fate of germline chromosome ends during somatic genome differentiation in the ciliate *Tetrahymena thermophila*. During sexual reproduction, a new somatic genome is created from a zygotic, germline-derived genome by extensive programmed DNA elimination events. It has been known for some time that the terminii of the germline chromosomes are eliminated, but the exact process and kinetics of the elimination events has not been thoroughly investigated. The authors first use germline-specific telomere probes to show that the loss of these chromosome ends occurs with similar timing as other DNA elimination events. By comparative analysis of the assembled germline and somatic genomes, the authors find the ends of each of the germline chromosomes are composed of few hundred kilobases of micronuclear limited sequences (MLS) that are removed starting around 14 hours after the start of conjugation, which initiates sexual development. They then develop an in-situ hybridization assay to track the fate of one end of chromosome 4 while simultaneously following the adjacent macronuclear destined sequence (MDS) retained in the new somatic genome. This allows the authors to more clearly show that these adjacent chromosomal segments are initially amplified in the developing genome before the terminal MLS is eliminated. Finally, they mutate the chromosome breakage sequence (CBS) that normally separates the MLS terminus from the adjacent MDS region as show that strains that develop with only one mutant chromosome can produce viable sexual progeny, but it appears that both the MLS and the MDS from the mutant chromosome are lost. If both chromosome copies have the CBS mutation, the cells arrest during development and do not eliminate many germline limited sequences and fail to produce viable progeny. Overall, this study provides many new insights into the fate of germline chromosome ends during somatic genome remodeling and suggests extensive coordination of different DNA elimination events in Tetrahymena.

Strengths:

Overall, the experiments were well executed with appropriate controls. The findings are generally robust. Importantly, the study provides several novel findings. First, the authors provide a fairly comprehensive characterization of the size of the MLS at the end of each germline chromosome. They also report on the highly repetitive composition of these chromosome terminii. Second, the authors develop a novel method to study the fate of chromosome terminii during development and use it conclusively track the elimination of these terminii. Third, the authors show that the elimination of these terminii appears to occur concurrently with most other DNA elimination events during somatic genome differentiation. And fourth, the authors show that failure to separate these eliminated sequences from the normally retained chromosome alters the fate of these adjacent MDS and loss of the cells ability to produce viable progeny. The authors initially hypothesized that DNA elimination may be blocked due to inappropriate silencing of genes in the MDS region when the CBS is mutant, but gene expression analysis showed that this is not the case.

Weaknesses:

After revising the manuscript based on the initial reviewers' critique, most weaknesses have been addressed. On weakness remaining is that since the authors only mutated the end of one germline chromosome, it is not clear whether the elimination of the MDS adjacent to the terminal MLS on chromosome 4 when the CBS is mutated is a general phenomenon, i.e. would happen at all chromosome ends, or is unique to the situation at Chromosome 4R. Knowing whether it is a general phenomenon or not would provide important insight into the authors findings. The authors did attempt to look at other chromosome ends, but technical limitations currently stymie this effort.

The other weakness is that it remains unclear how failure to carry out DNA elimination appears to induce a checkpoint during development, but this open question is not unique to this study.

Comments on revised version.

The authors have significantly improved the study. The addition of the RNA-seq analysis allowed these researchers to show that their initial hypothesis - that loss of a CBS leads to inappropriate gene silencing in the neighboring MDS region - appears not to be the case. I do not have further suggestions for the authors.

# Referee report: Reviewer #2 (Public review)

**DOI:** 10.7554/eLife.109351.3.sa2

Summary:

Mochizuki and colleagues investigated how the germline (MIC) telomere was removed during programmed genome rearrangement in the developing somatic nucleus (MAC). Using an optimized oligo-FISH procedure, the authors demonstrated that MIC telomeres were co-eliminated with a large region of MIC-limited sequences (MLS) demarcated on the opposite side by a sub-telomeric chromosome breakage site (CBS). This conclusion was corroborated by the latest assembly of the Tetrahymena MIC genome. They further employed CRISPR-Cas9 mutagenesis to disrupt a specific sub-telomeric CBS (4R-CBS). In the uniparental progeny (mutant X WT), DNA elimination of the sub-telomeric MLS was not affected, but the adjacent MAC-destined sequence (MDS) may be co-eliminated. However, in the biparental progeny (mutant X mutant), global DNA elimination was arrested, revealing previously unrecognized connections between chromosome breakage and DNA elimination. It also paves the way for future studies into the underlying molecular mechanisms. The work is rigorous, well-controlled, and offers important insights into how eukaryotic genomes demarcate genic regions (retained DNA) and regions derived from transposable element (TE; eliminated DNA) during differentiation. The identification of chromosome breakage sequences as a critical architectural element of the genome separating TE-derived regions from functional genes is a key conceptual contribution.

Strengths:

New method development: Oligo-FISH in Tetrahymena. This allows high-resolution visualization of critical genome rearrangement events during MIC-to-MAC differentiation. This method will be a very powerful tool in this area of study.

The conclusion is strongly supported by integrated analyses of PCR-based assays, as well as cytological, genomic, and transcriptomic data.

Rigorous genetic analysis of the role played by 4R-CBS in separating the fate of sub-telomeric MLS (elimination) and MDS (retention).

# Referee report: Reviewer #3 (Public review)

**DOI:** 10.7554/eLife.109351.3.sa3

Programmed DNA elimination (PDE) is a process that removes a substantial amount of genomic DNA during development. While it contradicts the genome constancy rule, an increasing number of organisms have been found to undergo PDE, indicating its potential biological function. Single-cell ciliates have been used as a prominent model system for studying PDE, providing important mechanistic insights into this process. Many of those studies have focused on the excision of internally eliminated sequences (IES) and the subsequent repair using non-homologous end joining (NHEJ). These studies have led to the identification of small RNAs that mark retained or eliminated regions and the transposons that generate double-strand breaks.

In this manuscript, Nagao and Mochizuki examined the other type of breaks in ciliates that are healed with telomere addition. They specifically focused on the sequences at the ends of the germline (MIC) chromosomes, which have received relatively less attention due to the technical challenges associated with the highly repetitive nature of the sequences. The authors used the Tetrahymena model and developed a set of new tools. They used a novel FISH strategy that enables the distinction between germline and somatic telomeres, as well as the retained and eliminated DNA near the chromosome ends. This allows them to track these sequences at the cellular level throughout the development process, where PDE occurs. They also analyzed the more comprehensive germline and somatic genomes and determined at the sequence level the loss of subtelomeric and telomere sequences at all chromosome ends. Their result is reminiscent of the PDE observed in nematodes, where all germline chromosome ends are removed and remodeled. Thus, the finding connects two independent PDE systems, a protozoan and a metazoan, and suggests the convergent evolution of chromosome end removal and remodeling in PDE.

The majority of sites (8/10) at the junctions of retained and eliminated DNA at the chromosome ends contain a chromosome breakage sequence (CBS). The authors created a set of mutants that modify the CBS at the ends of chromosome 4R. CBS regions are challenging for CRISPR due to their AT-rich sequences, making the creation of the 4R-CBS mutants a significant breakthrough. They used the FISH assay to determine if PDE still occurs in these mutant strains with compromised CBS. Surprisingly, they found that instead of blocking PDE, its adjacent retained DNA is now eliminated, suggesting a co-elimination event when the breakage is impaired. Furthermore, in biparental mutant crosses, no PDE occurred, and no viable progeny were produced, indicating that the removal of chromosome ends is crucial for proper PDE and sexual progeny development. Overall, the work demonstrates a critical role for 4R-CBS in separating retained and eliminated DNA.

# Author comment: Author response

**DOI:** 10.7554/eLife.109351.3.sa4

**Authors:** Kohei Nagao, Alix Lemoine, Tomoko Noto, Kazufumi Mochizuki

**Affiliations:** Centre National de la Recherche Scientifique Montpellier France; Centre National de la Recherche Scientifique Montpellier France; Centre National de la Recherche Scientifique Montpellier France; Centre National de la Recherche Scientifique Montpellier France

The following is the authors’ response to the original reviews.

(1) We bioinformatically examined the repeat compositions of MLSs (Figure 3B), which clearly indicated that all MLSs are composed of repetitive sequences to a much greater extent than the rest of the genome.

(2) We confirmed the blockage of chromosome breakage by the 4R-CBS mutations using a telomere-anchored PCR assay (Figure 5C-E).

(3) We examined the effect of the 4R-CBS mutations on the expression of genes encoded in 4R-MDS by RNA-seq (Figure 9). This analysis unexpectedly revealed that gene expression from 4R-MDS is not significantly affected in the mutants, allowing us to extend our discussion.

(4) We added two authors, Alix Lemoine and Tomoko Noto, who performed the experiments for these revisions.

**Public Reviews:**

**Reviewer #1 (Public review):**

Summary:

In this study, Nagao and Mochizuki examine the fate of germline chromosome ends during somatic genome differentiation in the ciliate *Tetrahymena thermophila*. During sexual reproduction, a new somatic genome is created from a zygotic, germline-derived genome by extensive programmed DNA elimination events. It has been known for some time that the termini of the germline chromosomes are eliminated, but the exact process and kinetics of the elimination events have not been thoroughly investigated. The authors first use germline-specific telomere probes to show that the loss of these chromosome ends occurs with similar timing as other DNA elimination events. By comparative analysis of the assembled germline and somatic genomes, the authors find that the ends of each of the germline chromosomes are composed of a few hundred kilobases of micronuclear limited sequences (MLS) that are removed starting around 14 hours after the start of conjugation, which initiates sexual development. They then develop an in situ hybridization assay to track the fate of one end of chromosome 4 while simultaneously following the adjacent macronuclear destined sequence (MDS) retained in the new somatic genome. This allows the authors to more clearly show that these adjacent chromosomal segments are initially amplified in the developing genome before the terminal MLS is eliminated. Finally, they mutate the chromosome breakage sequence (CBS) that normally separates the MLS terminus from the adjacent MDS region, to show that strains that develop with only one mutant chromosome can produce viable sexual progeny, but it appears that both the MLS and the MDS from the mutant chromosome are lost. If both chromosome copies have the CBS mutation, the cells arrest during development and do not eliminate many germline-limited sequences and fail to produce viable progeny. Overall, this study provides many new insights into the fate of germline chromosome ends during somatic genome remodeling and suggests extensive coordination of different DNA elimination events in Tetrahymena.

Strengths:

Overall, the experiments were well executed with appropriate controls. The findings are generally robust. Importantly, the study provides several novel findings. First, the authors provide a fairly comprehensive characterization of the size of the MLS at the end of each germline chromosome. I'm not sure whether this has been published elsewhere. Second, the authors develop a novel method to study the fate of chromosome termini during development and use it to conclusively track the elimination of these termini. Third, the authors show that the elimination of these termini appears to occur concurrently with most other DNA elimination events during somatic genome differentiation. And fourth, the authors show that failure to separate these eliminated sequences from the normally retained chromosome alters the fate of these adjacent MDS and the loss of the cells' ability to produce viable progeny.

Weaknesses:

It appears the authors did extensive analysis of the MLS chromosome ends, but did not provide too much information related to their composition. If this has not been published elsewhere, it would be useful to describe the proportion of unique and repetitive sequences and provide more information about the general composition of the chromosome ends. Such information would help the reader understand the nature of these MLS and how they may or may not differ from other eliminated sequences.

We now calculated the proportions of unique and repetitive sequences for each MLS, and these data are included in Figure 3B and described in the main text of the revised manuscript. A more comprehensive analysis of chromosome-end composition, including detailed characterization in the context of the complete MIC genome assembly, is beyond the scope of the current study and will be presented in a future publication.

Although the development of the novel FISH probes for large chromosome ends allowed for these novel discoveries, the signal in several images was visible, but often quite faint. I'm not sure there is anything the authors could do to improve the signal-to-noise ratio, but one needs to stare at the images carefully to understand the findings.

We have submitted higher-resolution images for the revised manuscript, which we believe much improve the visibility of faint signals.

One main weakness in the opinion of this reviewer is that the authors did very little to understand why, when a terminal MLS and the adjacent MDS fail to get separated because of failure in chromosome breakage, both segments are eliminated. The authors propose that possibly essential genes in the MDS get silenced, and the resulting lack of gene expression is the issue, but this and other possibilities were not tested. The study would provide more mechanistic insight if they had tried to assess whether the MDS on the CBS mutant chromosome becomes enriched in silencing modifications (e.g., H3K9me3). Alternatively, the authors could have examined changes in gene expression for some of the loci on the neighbouring MDS.

The 4R-CBS mutation causes two distinct defects that should be considered separately: (1) co-elimination of 4R-MLS and the adjacent 4R-MDS during uniparental transmission of the 4R-CBS mutation; and (2) a global block of DNA elimination during biparental transmission of the 4R-CBS mutation.

For the first defect, 4R-MLS and 4R-MDS may simply co-segregate into the nuclear compartment where DNA elimination occurs when the chromosome break that normally separates 4R-MLS from 4R-MDS is blocked. In this scenario, no additional process, such as spreading of scnRNA production, heterochromatin formation, or gene silencing, would be required to induce co-elimination. This point was not clearly stated in the previous manuscript, and we have now added a discussion of it to the revised manuscript.

The possibility of gene silencing within 4R-MDS was raised as a potential explanation for the second defect. To test this possibility, we performed RNA-seq analysis of wild-type and 4R-CBS mutant cells to determine whether gene expression from 4R-MDS is affected by mutations at 4R-CBS. Contrary to our expectations, we found that genes in 4R-MDS are not significantly down-regulated in 4R-CBS mutant cells compared with other genes. This result suggests that the DNA elimination defect in these cells cannot be explained by silencing of genes located within 4R-MDS. We have added these RNA-seq data to Figure 9 and described them in the Results section. We have also revised the Discussion to propose alternative possibilities that may guide future investigations.

The other main weakness is that since the authors only mutated the end of one germline chromosome, it is not clear whether the elimination of the MDS adjacent to the terminal MLS on chromosome 4 when the CBS is mutated is a general phenomenon, i.e., would happen at all chromosome ends, or is unique to the situation at Chromosome 4R. Knowing whether it is a general phenomenon or not would provide important insight into the authors' findings.

As was described in the manuscript, the short (CBS = 15 nt) target within AT-rich and repetitive regions prevent designing gRNAs specifically targeting some of the chromosome end CBSs. We tried to mutate the CBS sequences of the left end of the chromosome 3 (3L) and the left end of the chromosome 5 (5L) by the strategy we used to mutate 4R-CBS but failed. Therefore, to systematically mutate other chromosome-end CBSs, we need to establish a different strategy, such as combining template-based repairing to CRISPR-induced DSB. We have explained this technical limitation and stated that “Our data support a critical role for 4R-CBS in separating 4R-MLS from 4R-MDS, but it remains unclear whether all MIC chromosome ends are strictly CBS-dependent for their elimination.” in Discussion (Page 12).

**Reviewer #2 (Public review):**

Summary:

Nagao and Mochizuki investigated how the germline (MIC) telomere was removed during programmed genome rearrangement in the developing somatic nucleus (MAC). Using an optimized oligo-FISH procedure, the authors demonstrated that MIC telomeres were co-eliminated with a large region of MIC-limited sequences (MLS) demarcated on the opposite side by a sub-telomeric chromosome breakage site (CBS). This conclusion was corroborated by the latest assembly of the Tetrahymena MIC genome. They further employed CRISPR-Cas9 mutagenesis to disrupt a specific sub-telomeric CBS (4R-CBS). In uniparental progeny (mutant X WT), DNA elimination of the sub-telomeric MLS was not affected, but the adjacent MAC-destined sequence (MDS) may be co-eliminated. However, in biparental progeny (mutant X mutant), global DNA elimination was arrested, revealing previously unrecognized connections between chromosome breakage and DNA elimination. It also paves the way for future studies into the underlying molecular mechanisms. The work is rigorous, well-controlled, and offers important insights into how eukaryotic genomes demarcate genic regions (retained DNA) and regions derived from transposable elements (TE; eliminated DNA) during differentiation. The identification of chromosome breakage sequences as barriers preventing the spread of silencing (and ultimately, DNA elimination) from TE-derived regions into functional somatic genes is a key conceptual contribution.

Strengths:

New method development: Oligo-FISH in Tetrahymena. This allows high-resolution visualization of critical genome rearrangement events during MIC-to-MAC differentiation. This method will be a very powerful tool in this area of study.

Integration of cytological and genomic data. The conclusion is strongly supported by both analyses.

Rigorous genetic analysis of the role played by 4R-CBS in separating the fate of sub-telomeric MLS (elimination) and MDS (retention). DNA elimination in ciliates has long been regarded as an extreme form of gene silencing. Now, chromosome breakage sequences can be viewed as an extreme form of gene insulators.

Weaknesses:

The finding of global disruption of DNA elimination in 4R-CBS mutant progeny is highly intriguing, but it's mostly presented as a hypothesis in the Discussion. The authors propose that the failure to separate MLS from MDS allows aberrant heterochromatin spreading from the former into the latter, potentially silencing genes required for DNA elimination itself. While supported by prior literature on heterochromatin feedback loops, the specific targets silenced are not identified. While results from ChIP-seq and small RNA-seq can greatly strengthen the paper, the reviewer understands that direct molecular characterization may be beyond the scope of the current work.

As mentioned in our reply to Reviewer #1’s comment above, we performed RNA-seq on wild-type and 4R-CBS mutant cells at 13.5 hpm and 15 hpm and found that genes in 4R-MDS are not significantly downregulated in 4R-CBS mutant cells (Figure 9), suggesting that the DNA elimination defect in these cells cannot be explained by aberrant heterochromatin spreading. Therefore, the link between the chromosome break at 4R-CBS and general DNA elimination remains elusive and will be a very interesting subject for our future research. We have added these results and revised the discussion in the manuscript.

**Reviewer #3 (Public review):**

Programmed DNA elimination (PDE) is a process that removes a substantial amount of genomic DNA during development. While it contradicts the genome constancy rule, an increasing number of organisms have been found to undergo PDE, indicating its potential biological function. Single-cell ciliates have been used as a prominent model system for studying PDE, providing important mechanistic insights into this process. Many of those studies have focused on the excision of internally eliminated sequences (IES) and the subsequent repair using non-homologous end joining (NHEJ). These studies have led to the identification of small RNAs that mark retained or eliminated regions and the transposons that generate double-strand breaks.

In this manuscript, Nagao and Mochizuki examined the other type of breaks in ciliates that were healed with telomere addition. They specifically focused on the sequences at the ends of the germline (MIC) chromosomes, which have received relatively less attention due to the technical challenges associated with the highly repetitive nature of the sequences. The authors used the Tetrahymena model and developed a set of new tools. They used a novel FISH strategy that enables the distinction between germline and somatic telomeres, as well as the retained and eliminated DNA near the chromosome ends. This allows them to track these sequences at the cellular level throughout the development process, where PDE occurs. They also analyzed the more comprehensive germline and somatic genomes and determined at the sequence level the loss of subtelomeric and telomere sequences at all chromosome ends. Their result is reminiscent of the PDE observed in nematodes, where all germline chromosome ends are removed and remodeled. Thus, the finding connects two independent PDE systems, a protozoan and a metazoan, and suggests the convergent evolution of chromosome end removal and remodeling in PDE.

The majority of sites (8/10) at the junctions of retained and eliminated DNA at the chromosome ends contain a chromosome breakage sequence (CBS). The authors created a set of mutants that modify the CBS at the ends of chromosome 4R. CBS regions are challenging for CRISPR due to their AT-rich sequences, making the creation of the 4R-CBS mutants a significant breakthrough. They used the FISH assay to determine if PDE still occurs in these mutant strains with compromised CBS. Surprisingly, they found that instead of blocking PDE, its adjacent retained DNA is now eliminated, suggesting a co-elimination event when the breakage is impaired. Furthermore, in biparental mutant crosses, no PDE occurred, and no viable progeny were produced, indicating that the removal of chromosome ends is crucial for proper PDE and sexual progeny development. Overall, the work demonstrates a critical role for 4R-CBS in separating retained and eliminated DNA.

We appreciate Reviewer 3’s assessment.

**Recommendations for the authors:**

**Reviewing Editor Comments:**

All reviewers agree that this study makes an important contribution to the field; however, they also offered several suggestions for how the manuscript could be improved. In particular, we draw your attention to the comments from Reviewer #1, who suggests that the manuscript could benefit from additional information on the general composition of germline chromosome ends, where available.

As noted in our response to Reviewer #1 in the Public Reviews above, we have included an analysis of the fraction of repetitive sequences for each MLS as Figure 3B in the revised manuscript, highlighting the highly repetitive nature of MLSs compared with the rest of the genome.

**Reviewer #1 (Recommendations for the authors):**

As mentioned in the weaknesses section, the authors could provide more information regarding the nature of the sequences that make up the terminal MLS. There have been reports that these are highly repetitive; is that the case? Also, did the authors identify common repeats that are not internal to mic chromosomes that could be used to track all terminal segments of the five chromosomes? This would complement their mic-telomere probe.

As noted in our response to Reviewer #1’s Public Review above, we have added an analysis of the fraction of repetitive sequences for each MLS as Figure 3B in the revised manuscript, which confirms that MLSs are highly repetitive.

Apart from the moderately conserved Telomere Associated Sequence (TAS), described by Kirk and Blackburn (1995) and of unknown function, we were unable to identify any obvious shared repeats unique to MLSs that could support the development of pan-MLS-specific probes.

One major weakness is that the authors did little to determine the cause of the elimination of the adjacent MDS along the 4R-MLS when the CBS was mutated. It would really improve the study if the authors could show that:

(1) Gene expression of genes on the MDS is reduced in 4r-CBS mutant progeny.

(2) Heterochromatin modifications are unexpectedly acquired on the MDS in mutants relative to wild-type chromosomes.

(3) Do scnRNA specific to the MDS region appear in the mutant progeny during development, but not in wild-type crosses?

Any data that would help support the authors' hypothesis regarding how the MDS region is eliminated when the CBS is mutant would definitely strengthen the conclusions of the study.

As noted in our response to Reviewer #1’s Public Review above, we performed RNA-seq on wild-type and 4R-CBS mutant cells at 13.5 hpm and 15 hpm. Our analysis showed that genes within the 4R-MDS are not significantly downregulated in 4R-CBS mutant cells (Figure 9), suggesting that the DNA elimination defect in these cells cannot be attributed to aberrant heterochromatin spreading. Therefore, the connection between the chromosome break at 4R-CBS and general DNA elimination remains unclear and represents an important avenue for future investigation. We have incorporated these results and revised the discussion accordingly in the updated manuscript.

The other main weakness is that by mutating the CBS of only one chromosome arm, one can't know whether the loss of the MDS with the MLS in the mutants is generalizable for all chromosome arms or is unique to 4R. The authors noted that they were unable to make any other mutated CBSs. Another way to try to get to this question is to try to rescue the mutant by inserting a new CBS into the 4R arm such that some MLS remains linked to the 4R-MDS and see whether removing the mic telomere is the issue, or would a block of MLS attached to the 4R-MDS be sufficient to cause its elimination. I'm not sure where to exactly put the new CBS, but worth thinking about.

To introduce a new CBS into 4R-MLS, we would need to insert a CBS-containing construct into the MIC by homologous recombination during conjugation and then select engineered transformants using a drug resistance marker expressed from the derived MAC. However, because 4R-MLS is still eliminated in the progeny of 4R-CBS mutants, the introduced marker would be lost from the MAC even if homologous recombination were successful. Therefore, although the strategy suggested by this reviewer is very interesting, several technical innovations are required to make such experiments feasible, leaving this approach for a future project.

It seems somewhat curious that the mutation of the CBS completely blocks nuclear development. In Paramecium, the failure to complete internal DNA elimination events can lead to alternative telomere addition. The caveat being that, in Paramecium, telomere addition appears more promiscuous than in Tetrahymena. It would be helpful to know how absolute the failure to produce progeny is in these mutants. Is it zero progeny in 10^6^, 10^7^, 10^8^ ..... mated cells? Can the authors provide a possible lowest possible frequency?

The viability tests were performed using bulk mating of 2.5 × 10^4^ cells for each cross. Because ~70-80% of mating pairs complete the conjugation process and produce exconjugants under our standard culture conditions, and because we did not detect any 6-mp-resistant progeny from MUT × MUT crosses, we estimate that the probability of obtaining viable progeny in these crosses was less than 1 progeny per ~2 × 10^4^ mating pairs. The number of cells used for the viability assay is described in the “Viability Test of Sexual Progeny” section of Materials and methods and the estimated frequency of progeny production from the mutants has been mentioned in Results section in the revised manuscript.

The one implication of the study is that chromosome breakage and DNA elimination, two different events, are coupled. In most mutants that block scnRNA-directed DNA elimination, both IES excision and chromosome breakage occur. In the study by McDaniel, SL. et al (2016). DRH1, a p68-related RNA helicase, is required for chromosome breakage in Tetrahymena. Biology Open pii: bio.021576. doi: 10.1242/bio.021576, germline knockouts of DRH1 could complete IES excision, but not chromosome breakage, indicating that the processes can be uncoupled. It may be useful for the authors to discuss this previous work in relation to their finding that failure in chromosome breakage can lead to DNA elimination of neighboring sequences.

So far, *DRH1* is the only gene reported to be required for chromosome breakage without affecting DNA elimination in *Tetrahymena*. However, McDaniel SL et al. (2016) examined chromosome breakage at only two CBSs (distinct from 4R-CBS), and thus it remains unclear how broadly chromosome breakage, including that at 4R-CBS, is affected in the absence of *DRH1*. In addition, McDaniel SL et al. (2016) assessed DNA elimination at three different IESs using PCR, whereas our study examined elimination of the repetitive Tlr1 transposon using FISH. Therefore, without further analysis of the similarities and differences in chromosome breakage and DNA elimination phenotypes between *DRH1* knockout cells and 4R-CBS mutants, it is difficult to draw meaningful conclusions. Accordingly, we have limited ourselves to stating the following in the Discussion of the revised manuscript: “Moreover, chromosome breakage can be inhibited without disrupting DNA elimination, as shown in cells lacking zygotic expression of the p68-like RNA helicase Drh1 (McDaniel et al., 2016).”

Minor corrections:

Page 7, line 3: the text "......inducing chromosome break" should either be "......inducing chromosome breaks" or "......inducing a chromosome break".

Corrected as “inducing a chromosome break”.

Page 13, line 13: "......large block...." should be "......large blocks......".

Corrected as suggested.

**Reviewer #2 (Recommendations for the authors):**

The authors can experimentally validate that chromosome breakage at 4R-CBS is indeed disrupted by the mutations. A PCR-based assay testing de novo telomere addition is a standard tool. In addition, MLS-linked telomere should only appear transiently during conjugation in WT cells.

Because it was previously unknown whether de novo telomere addition occurs at the ends of MLSs upon chromosome breakage, we tested this using a PCR-based assay. We detected telomere-added chromosome ends of 4R-MLS and 3L-MLS, which were undetectable until 10.5 hpm, appeared at 12 hpm, and gradually decreased by 18 hpm in wild-type cells (WT × WT cross). Importantly, the appearance of the telomere-added 4R-MLS end, but not the 3L-MLS end, was blocked in 4R-CBS mutants (Mut × Mut crosses), strongly supporting that the 4R-CBS mutations specifically disrupt chromosome breakage at 4R-CBS. These new data are shown in Figure 5C–E and described in the Results section.

The high FISH background during conjugation may be caused by the abundant presence of dsRNA, which is resistant to RNase A treatment but may be degraded by RNase III.

The high FISH background was observed in the parental MAC at 9 and 12 hpm (Figure 2, 4, and S2) where dsRNA accumulation was not detected in the previous studies (Woo et al. 2016; Shehzada et al. 2024). In contrast, the MIC at 3 hpm and the new MAC at 9 and 12 hpm, where strong dsRNA accumulation was detected, showed much weaker background FISH signals (Figure 2, 4, and S2). Therefore, we believe that dsRNA is not the main cause of the high FISH background.

It is likely that the long MIC telomere is treated as IES and targeted for DNA elimination. Indeed, telomere-specific scnRNA is abundantly produced during conjugation (http://www.ncbi.nlm.nih.gov/pubmed/19460867).

We have cited the suggested literature and the following description has been added in Discussion to relate the reported telomere-derived scnRNAs to the abundant scnRNAs produced from MIC chromosomal ends: “In addition, telomere-complementary scnRNAs were reported to be produced specifically during conjugation (Cao et al. 2009).”

Global disruption of DNA elimination may be a direct effect (DNA excision machinery affected) or indirect (unrepaired DSB and checkpoint activation).

It has been reported that unrepaired DSBs caused by loss of Ku80 (Tku80) do not block DNA elimination in *Tetrahymena* (Lin et al. 2012). Therefore, checkpoint activation by unrepaired DSBs, if it occurs, is unlikely to explain the DNA elimination defect observed in the progeny of 4R-CBS mutants. Nonetheless, this direct-versus-indirect issue would be relevant when considering whether disruption of specific 4R-MDS-encoded genes in 4R-CBS mutants could cause the DNA elimination defect. Our new RNA-seq analysis, however, suggests that this possibility is unlikely. Therefore, we did not add further discussion of this direct-versus-indirect issue.

Minor points:

The zoom-in boxes in most images are barely visible.

We have modified the zoom-in boxes to make them clearer.

Page 13: scnRNA precursors (Cai et al., 2025) (Cai et al., in press). Is it one paper or two?

They are two papers and the latter was published reacently. We have updated the citation.

**Reviewer #3 (Recommendations for the authors):**

The manuscript is well-written, with clear data, thoughtful discussion, and concise presentation. I have only a few minor comments below.

For Figure 4 and others, the right panel shows the stats and percentages, with positive and negative labels. It's a bit confusing at first glance. I think it can be clarified what positive and negative mean in the legend.

The legends of Figure 4, Figure 6 and Supplementary Figure S2, have been modified as “The presence (Positive) or absence (Negative) of the 4R-MLS FISH signal in new MAC (An) in 50 cells per time point was examined.”

The quality of the FISH images is low at their current resolution. It is difficult to get a clear view.

In the initial version, some images were in low resolution when we combined them into a single pdf file for review. In the revised manuscript, the images have been replaced with high-resolution images.

The co-elimination of neighboring 4R-MDS when 4R-CBS is mutated, can this be viewed as a fail-safe mechanism to ensure the elimination of the chromosome ends? Regardless, the result begs the question of the significance of end removal and remodeling of PDE. Some speculations in the discussion might be helpful.

Because the neighboring 4R-MDS contains approximately 100 predicted genes, its co-elimination would likely be too risky to evolve as a fail-safe mechanism for ensuring chromosome-end elimination in every generation. Instead, we interpret this as an erroneous process that can still be compensated for through endoreplication of the remaining, normally processed 4R-MDS from the non-mutated copy.

We further speculate that the connection between chromosome breakage at 4R-CBS and the essential PDE process may serve as an evolutionary pressure to preserve the 4R-CBS locus in a chromosome breakage-competent state. We have added the following discussion to the revised manuscript (Page 15): “The observed link between chromosome breakage at 4R-CBS and the essential DNA elimination process may reflect the biological significance of MLSs and the importance of their removal from the MAC. Coupling these processes may have evolved as a mechanism to ensure that only functional chromosome-end CBS loci are preferentially transmitted to future generations.”

Figure 1, legend, line 3, "the sexual reproduction process", do you mean "the sexual reproduction proceeds or initiates"?

We meant “conjugation” = “the sexual reproduction process”. To make this clearer, we have revised the legend as “conjugation, which is the sexual reproduction process of *Tetrahymena*”.
